# Did the extended coverage policy contribute to alleviating socioeconomic inequality in untreated dental caries of both children and adolescents in South Korea?

**DOI:** 10.1186/s12903-020-01112-8

**Published:** 2020-04-22

**Authors:** Bo-Mi Shin, Se-Hwan Jung, Myoung-Hee Kim, Jae-In Ryu

**Affiliations:** 1 Department of Dental Hygiene, College of Dentistry, Gangneung-Wonju National University, 120 Gangneungdaehag-ro, Gangneung City, Gangwon Province 25457 South Korea; 2 Department of Preventive and Public Health Dentistry, College of Dentistry, Gangneung-Wonju National University, 120 Gangneungdaehag-ro, Gangneung City, Gangwon Province 25457 South Korea; 3 Center for Health Equity Research, People’s Health Institute, 36 Sadangro 13-gil, 2nd floor, Dongjak-gu, Seoul, 07004 South Korea; 4 Department of Preventive and Social Dentistry, College of Dentistry, Kyung Hee University, 26 Kyungheedae-ro, Dongdaemun-gu, Seoul, 02447 South Korea

**Keywords:** Children and adolescent health, Socioeconomic factors, Inequality, Service provision

## Abstract

**Background:**

Dental sealants have been covered by the National Health Insurance Service (NHIS) since December 2009 in South Korea. This study aims to determine whether the socioeconomic inequality in untreated dental caries decreased after implementing the extended coverage policy for dental sealant.

**Methods:**

The data were derived from the fourth (2007–2009) and sixth (2013–2015) waves of the Korean National Health and Nutrition Examination Survey (KNHANES) conducted by the Korea Centers for Disease Control and Prevention (KCDC). Dental caries and sealant experience by income quartiles were tested using the Rao-Scott chi-squared test. In order to examine socioeconomic inequalities and their trends over time, the prevalence ratios (PRs), slope index of inequality (SII), and relative index of inequality (RII) were estimated for each wave and age group. All analyses were conducted using SAS version 9.3.

**Results:**

The adjusted PRs of untreated dental caries and sealants in the poorest in the aged 6–11 group were significantly higher and lower, respectively, compared to the most affluent quartile group for the fourth wave; however, all significant differences disappeared for the sixth wave, after the sealant coverage. The gap between the lowest and the highest was similar for the aged 12–18 group but it widened in the untreated dental caries even after the sealant coverage. The statistical significance of the PRs was maintained at the sixth wave for both caries and sealants. Children showed decreases in both SII and RII over time so its significance disappeared. The SII among adolescents decreased over time but the RII of untreated dental caries increased.

**Conclusions:**

This study found that the NHIS coverage expansion of dental care had a positive effect on overall status in dental health among children and adolescents. However, younger children benefited more in terms of inequalities. Our findings indicate that strategies to enhance access to preventive dental services should consider the differential effects for the vulnerable population in terms of socioeconomic status and age from the beginning stage of the policy.

## Background

Dental caries is the tenth most prevalent disease in the world, representing a significant burden of disease, especially in children [1, 2]. More than a quarter of the global population, almost 2.4 billion, suffer from untreated dental caries. Moreover, it is very expensive, as shown to be the fourth most expensive disease for effective treatment [3]. The bigger issue is that oral health conditions often indicate socioeconomic inequalities. A previous systematic review revealed that an individual’s socioeconomic position (SEP), such as parental or own education, occupation, and family income, was significantly associated with dental caries [4]. Those with higher SEP had a lower risk of having or having experienced dental caries.

Preventive care is the most effective way to minimize both the health and financial burdens of untreated caries. Dental sealants and fluoride applications are common and effective methods for preventing dental caries [5]. In the United States, school-based programs provide sealants to children, especially for subsidized meal program recipients [6]. Students who did not receive sealant treatment showed a two to three time higher mean numbers of decayed or filled first molars than students who did [7]. They suggested that universal caries prevention could reduce the incidence of dental caries by up to 80%, leading to savings on dental expenses [8]. A Chinese cohort study also showed that the hazard ratio for dental caries was 0.6 times lower in the treatment group received sealants compared to the control group [9].

However, a Dutch study showed undesirable outcomes regarding socioeconomic inequalities in dental health in spite of the expansion of dental care coverage. The Netherlands’ government offers free dental services for individuals under 19 years old and most of the participants visited dental professionals almost every year. Even after the expansion children in low SEP still had 1.5 times higher risk of having caries than children in higher SEP. This gap persisted beyond childhood and continued until they were young adults [10]. Similar results were found in Sweden, where the government provides free dental services for children and adolescents. The children in lower SEP had two to five times higher odds ratios of having dental caries than the higher SEP group, controlling for covariates such as ethnicity, wealth, parental education, and employment [11]. A meta-analysis also reported that developed countries with relatively equal income distribution showed more unequal caries experience, compared to developing countries with unequal income distribution [4]. These inconsistent findings suggest that there must be diverse factors related to the prevalence or experience of dental caries and that similar factors might have different effects, depending upon countries’ health care systems and their economic development status [12–14].

In South Korea (hereafter, Korea), dental sealants have been covered by the National Health Insurance Service (NHIS) since December 2009 [15]. In the beginning, the policy offered limited coverage, only the first molars of children aged 6 to 14. In May 2013, the coverage was expanded up to the second molars of children up to 18 years old. Accordingly, the proportion of children aged 6–14 years who received dental sealant increased encouragingly, from 28.7 to 34.9% [16]. A Korean study reported that after the policy implementation, dental sealant increased and untreated caries decreased, especially among lower SEP groups [17]. Many existing studies have suggested that equal access to dental treatment is essential to improve the dental status of all [18]. However, there is a gap in the literature concerning whether these governmental policies contribute to alleviating inequality in children’s oral health [19].

This study aims to determine whether the socioeconomic inequality in untreated dental caries decreased after implementing the extended coverage policy for dental sealant in South Korea.

## Methods

### Study design and participants

The data were derived from the fourth (2007–2009) and sixth (2013–2015) waves of the Korean National Health and Nutrition Examination Survey (KNHANES) conducted by the Korea Centers for Disease Control and Prevention (KCDC). The KNHANES is a repeated cross-sectional survey on a representative national sample that is based on multi-stage clustered probability samples from Korean households representing the civilian non-institutionalized population aged 1 year and older [20]. Each year, 192 sampling units and 20 households per primary sampling unit are selected according to location, age, and gender, yielding approximately 10,000 subjects in the age group.

The research team sent the written notice for examinations and surveys to selected household members with a brief introduction. Only the members who agreed to participate with written ethical approval and consent forms, acquired from themselves or their guardians if they were under 14-year-olds, were included in this study,

The survey consists of a health examination, a health interview, and a nutrition survey. It also includes an oral health examination by a dentist and a questionnaire interview regarding oral health behaviors. The KNHANES is certified and used as the national statistics by the Korea Department of Statistics. The raw data of the KNHANES are publicly available on their official website [21]. For the samples used in this study, the response rates were 78.4% in the fourth [22–24] and 78.3% in the sixth-wave [25–27], respectively. A total of 50,405 individuals participated, with informed consent. This study analyzed data from 7410 participants aged 6–18 years old (4353 in the fourth and 2915 in the sixth-wave) after excluding 1040 individuals due to missing information about oral health status, household income, and dental health behaviors. The study participants were divided into two groups: children aged 6–11 years old and adolescents aged 12–18 years old. In South Korea, the children enter the elementary school at 6, the middle school at 12, and high school at 15. The children and adolescents were separated because the coverage of the dental sealant in South Korea started from the children aged 6 to 14 then extended up to 18-year-old adolescents later. They were expected to show different patterns in untreated dental caries by the policy change. The Institutional Review Board of Gangneung-Wonju National University reviewed and approved this study (GWNUIRB-2016-07).

### Data variables

We set the outcome variables as dental caries and sealant experience, evaluated by dentists based on WHO criteria, who had completed the calibration training program and carried out clinical oral examinations in the mobile health examination centers [28]. For the dentist calibration training, the dental status measures were validated by comparing it with a reference dentist. As inter-examiner reliabilities, the mean Kappa values for tooth status were 0.711 to 0.919 by 47 dentists in the fourth [29–31] and 0.892 to 0.939 by 90 dentists in the sixth-wave [32–34]. Socioeconomic variables included household monthly income, equivalized for household size (equivalent household income = total household income ÷ [household size]^0.5^) and categorized into four quartiles. Dental health behaviors were considered as potential mediators to the relationship between income and dental health, while age and gender were considered as confounders. The frequency of tooth brushing (FTB) was categorized into two groups: brushing less than twice a day vs. two or more times a day. Regular dental check-ups (RDC) was classified as yes or no, asking whether they had visited the dentist for a regular check-up without any symptoms during the year prior to the interview.

### Data analysis

We used a complex sample analysis method to consider complex sample designs, including primary sampling units, stratification, and sample weights. The oral health conditions by income quartiles were tested using the Rao-Scott chi-squared test (Proc Surveyfreq). In order to examine socioeconomic inequalities and their trends over time, the prevalence ratios (PRs), slope index of inequality (SII), and relative index of inequality (RII) were estimated for each wave and age group. All the analyses were done twice as Model 1 adjusted with age and gender and Model 2 adjusted with age, gender, and dental health behavior such as FTB (frequency of tooth brushing) and RDC (regular dental check-up). The PRs and 95% confidence intervals (CIs) were estimated to assess the association between household income quartiles and untreated dental caries or sealant experiences in Korean children. SII represents the absolute difference in values between the lowest and the highest ends of SEP [35], while RII represents the ratio of the prevalence between the highest and lowest ends [35–37]. SII and RII were calculated using the relative income position indicator on the cumulative distribution of age-group specific equivalized income. This relative position indicator is a value between 0 and 1, allocated by calculating the mid-point of the relative position in the cumulative population distribution in income group, and was used as an independent variable in the log-binomial regression analyses. The LINK IDENTITY option of Proc GENMOD in SAS was used to calculate SII, while the LINK LOG option was used for calculating RII and PR [38, 39]. All analyses were conducted using SAS version 9.3 (SAS Institute Inc., Cary, NC, USA). All the models converged within the availability of the calculations of PR, SII, and RII.

## Results

The general characteristics of the study participants are shown in Table 1. The gender and income distribution of both age groups were similar at the fourth and sixth wave, respectively.

**Table 1:** Study Sample Characteristics: *N* (%)

Variables	Children aged 6–11			Adolescents aged 12–18		
	2007–09	2013–15	*P*-value	2007–09	2013–15	*P*-value
Total	2240 (100)	1449 (100)		2113 (100)	1466 (100)	
Gender						
Male	1167 (52.1)	758 (51.9)	0.937	1113 (53.7)	812 (52.1)	0.572
Female	1073 (47.9)	691 (48.1)		1000 (46.3)	718 (47.9)	
Income level						
I (lowest)	553 (24.9)	347 (23.7)	0.671	524 (24.8)	348 (25.0)	0.872
II	544 (23.9)	359 (26.7)		518 (24.8)	363 (25.1)	
III	568 (25.3)	368 (25.2)		522 (24.8)	391 (25.7)	
IV (highest)	575 (25.9)	375 (24.4)		549 (25.6)	364 (24.2)	
FTB						
< 2	308 (13.8)	117 (7.9)	< 0.001	261 (12.3)	108 (7.7)	< 0.001
≥ 2	1932 (86.2)	1332 (92.1)		1852 (87.7)	1358 (92.3)	
RDC						
No	755 (32.2)	427 (28.4)	0.116	1124 (53.4)	820 (57.3)	0.025
Yes	1485 (67.8)	1022 (71.6)		989 (46.6)	646 (42.7)	

*P*-values were obtained from complex samples crosstabs: Rao-Scott chi-squared test

Values are presented by weighted prevalence % (95% CI)

FTB (frequency of tooth brushing) and RDC (regular dental check-ups)

Table 2 shows the prevalence of untreated dental caries and sealant by gender and household income. The prevalence of untreated dental caries decreased in both age groups and all the income groups over time, except for the highest income quartile of the children. In contrast, the sealant prevalence increased over time across all ages and income groups. For the fourth wave, there was a significant socioeconomic inequality in untreated dental caries and sealant prevalence in both age groups; however, for the sixth wave, there was no significant difference among the aged 6–11 group.

**Table 2:** Prevalence rates (95% CI) of untreated dental caries and sealant by income level

Variables	Children aged 6–11		Difference	Adolescents aged 12–18		Difference
	2007–09	2013–15		2007–09	2013–15	
**Untreated dental caries**						
Total	8.7 (7.3–10.1)	5.7 (4.3–7.1)	−3.0	32.7 (30.1–35.3)	23.7 (20.7–26.7)	−9.0
Gender						
Male	8.9 (6.9–10.9)	5.3 (3.5–7.2)	−3.5	31.6 (28.3–35.0)	24.7 (21.0–28.4)	−6.9
Female	8.5 (6.6–10.4)	6.1 (4.2–8.1)	−2.4	34.0 (30.4–37.6)	22.6 (18.7–26.6)	−11.4
*P*-value	0.795	0.536		0.315	0.386	
Income level						
I (lowest)	11.9 (8.8–14.9)	5.7 (3.2–8.2)	−6.2	42.0 (36.5–47.4)	32.0 (25.9–38.1)	−10.0
II	10.5 (7.5–13.6)	7.1 (4.0–10.1)	−3.5	33.3 (28.5–38.1)	20.6 (15.3–25.8)	−12.7
III	7.4 (4.9–9.8)	3.2 (1.4–5.0)	−4.1	27.6 (22.8–32.3)	22.3 (17.2–27.4)	−5.2
IV (highest)	5.2 (3.2–7.1)	6.8 (3.6–10.0)	1.6	28.2 (23.8–32.7)	19.9 (14.1–25.6)	−8.4
*P*-value	**0.001**	0.152		**< 0.001**	**0.006**	
FTB						
< 2	14.8 (10.4–19.3)	9.6 (3.6–15.5)	−5.3	30.7 (23.9–37.4)	31.0 (21.2–40.7)	0.3
≥ 2	7.7 (6.2–9.2)	5.4 (4.0–6.8)	−2.3	33.0 (30.4–35.7)	23.1 (20.0–26.2)	−9.9
*P*-value	**0.001**	0.091		0.513	0.095	
RDC						
No	10.0 (7.6–12.3)	5.1 (2.8–7.3)	−4.9	34.2 (30.8–37.6)	28.1 (24.0–32.2)	−6.1
Yes	8.1 (6.3–9.8)	6.0 (4.2–7.7)	−2.1	31.0 (27.5–34.5)	17.8 (14.5–21.1)	−13.2
*P*-value	0.192	0.553		0.166	**< 0.001**	
**Sealants**						
Total	30.6 (28.0–33.3)	40.7 (37.5–44.0)	10.1	26.8 (24.2–29.3)	37.0 (33.8–40.2)	10.2
Gender						
Male	29.6 (26.3–32.9)	40.3 (36.2–44.4)	10.7	26.1 (23.1–29.2)	36.4 (32.5–40.3)	10.3
Female	31.7 (28.2–35.2)	41.2 (36.9–45.6)	9.5	27.5 (23.9–31.1)	37.6 (33.3–41.9)	10.1
*P*-value	0.320	0.737		0.539	0.651	
Income level						
I (lowest)	24.5 (20.2–28.7)	40.1 (34.1–46.1)	15.6	16.9 (13.0–20.7)	27.8 (22.5–33.0)	10.9
II	31.7 (26.9–36.4)	37.5 (31.6–43.4)	5.8	24.4 (19.7–29.0)	38.5 (32.3–44.6)	14.1
III	30.9 (26.0–35.8)	42.7 (36.8–48.6)	11.8	29.6 (25.1–34.1)	37.3 (31.8–42.7)	7.7
IV (highest)	35.3 (30.5–40.1)	42.9 (36.7–49.2)	7.6	35.9 (30.9–40.9)	44.6 (38.3–51.0)	8.7
*P*-value	**0.008**	0.522		**< 0.001**	**0.001**	
FTB						
< 2	24.6 (19.2–30.0)	35.1 (25.7–44.5)	10.5	20.4 (15.1–25.7)	35.6 (25.5–45.8)	15.2
≥ 2	31.6 (28.8–34.4)	41.2 (37.8–44.6)	9.7	27.7 (25.0–30.3)	37.1 (33.8–40.4)	9.4
*P*-value	**0.024**	0.240		**0.020**	0.789	
RDC						
No	24.8 (20.7–28.9)	36.1 (30.3–41.9)	11.3	21.9 (18.8–25.0)	33.4 (29.5–37.3)	11.6
Yes	33.4 (30.3–36.5)	42.6 (38.9–46.3)	9.2	32.4 (28.7–36.1)	41.7 (37.2–46.2)	9.4
*P*-value	**0.001**	0.062		**< 0.001**	**0.003**	

*P*-values were obtained from complex samples crosstabs: Rao-Scott chi-squared test

Values are presented by weighted prevalence % (95% CI)

FTB (frequency of tooth brushing) and RDC (regular dental check-ups)

Table 3 shows the adjusted PRs of untreated dental caries and sealants by income quartiles for the fourth and sixth waves. The adjusted PRs of untreated dental caries and sealants in the lowest income quartile in the aged 6–11 group were significantly higher or lower, respectively, compared to the highest quartile group for the fourth wave; however, all significant differences disappeared for the sixth wave, after the sealant coverage. The gap between the lowest and the highest was similar for the aged 12–18 group but it widened in the untreated dental caries even after the sealant coverage. The statistical significance of the PRs was maintained at the sixth wave for both caries and sealants.

**Table 3:** Changes in prevalence ratios (PRs) (95% CI) of untreated dental caries and sealant by income level

	Children aged 6–11				Adolescents aged 12–18			
	2007–09	2013–15			2007–09		2013–15	
	Model 1^**a**^	Model 2^**b**^	Model 1^**a**^	Model 2^**b**^	Model 1^**a**^	Model 2^**b**^	Model 1^**a**^	Model 2^**b**^
**Untreated dental caries**								
Income level								
I (lowest)	2.29 (1.51, 3.50)^***^	2.11 (1.38, 3.23)^***^	1.12 (0.64, 1.96)^NS^	1.10(0.62, 1.93)^NS^	1.47 (1.24, 1.74)^***^	1.44 (1.22, 1.71)^***^	1.77 (1.37, 2.32)^***^	1.67 (1.28, 2.18)^***^
II	2.18 (1.43, 3.34)^***^	2.14 (1.40, 3.28)^***^	0.91 (0.51, 1.63)^NS^	0.91 (0.51, 1.63)^NS^	1.12 (0.93, 1.35)^NS^	1.11 (0.92, 1.33)^NS^	1.19 (0.88, 1.59)^NS^	1.15 (0.85, 1.54)^NS^
III	1.44 (0.91, 2.27)^NS^	1.44 (0.91, 2.27)^NS^	0.66 (0.35, 1.25)^NS^	0.67 (0.36, 1.27)^NS^	0.96 (0.79, 1.17)^NS^	0.96 (0.79, 1.17)^NS^	1.17 (0.87, 1.56)^NS^	1.16 (0.87, 1.55)^NS^
IV (highest)	1.00	1.00	1.00	1.00	1.00	1.00	1.00	1.00
**Sealants**								
Income level								
I (lowest)	0.70 (0.59, 0.85)^***^	0.73 (0.61, 0.88)^***^	0.90 (0.76, 1.06)^NS^	0.92 (0.78, 1.09)^NS^	0.54 (0.45, 0.66)^***^	0.57 (0.47, 0.70)^***^	0.70 (0.58, 0.85)^***^	0.70 (0.58, 0.86)^***^
II	0.88 (0.74, 1.03)^NS^	0.89 (0.75, 1.04)^NS^	0.89 (0.75, 1.04)^NS^	0.90 (0.76, 1.06)^NS^	0.68 (0.57, 0.82)^***^	0.71 (0.59, 0.84)^***^	0.86 (0.73, 1.03)^NS^	0.87 (0.73, 1.03)^NS^
III	0.89 (0.76, 1.05)^NS^	0.90 (0.76, 1.05)^NS^	1.01 (0.86, 1.18)^NS^	1.01 (0.87, 1.18)^NS^	0.85 (0.72, 1.00)^*^	0.85 (0.73, 1.00)^NS^	0.86 (0.73, 1.02)^NS^	0.86 (0.73, 1.01)^NS^
IV (highest)	1.00	1.00	1.00	1.00	1.00	1.00	1.00	1.00

^a^ Adjusted for age, gender

^b^ Adjusted for age, gender, FTB (frequency of tooth brushing), and RDC (regular dental check-up)

* *P* < 0.05, **P < 0.01, *** *P* < 0.001

Table 4 shows the absolute and relative inequalities in untreated dental caries and sealant prevalence in both age groups by income quartiles. The changing pattern of SII and RII for sealant and untreated dental caries prevalence were different in two age groups between the fourth and the sixth wave. Children showed alleviates in both SII and RII over time and its significance disappeared. The SII among adolescents decreased over time, for example, from 16.4 (95% CI: 9.6–23.2) to 13.9 (95% CI: 6.5–21.2) as well. However, the RII of untreated dental caries increased from 1.7 (95% CI: 1.4–2.2) to 2.1 (95% CI: 1.5–2.9) exceptionally.

**Table 4:** Absolute and Relative socio-economic inequalities (95% CI) of untreated dental caries and sealant by income level

	Children aged 6–11				Adolescents aged 12–18			
	2007–09		2013–15		2007–09		2013–15	
	Model 1^**a**^	Model 2^**b**^	Model 1^**a**^	Model 2^**b**^	Model 1^**a**^	Model 2^**b**^	Model 1^**a**^	Model 2^**b**^
**Untreated dental caries**								
SII	7.3 (3.3, 11.3) ^***^	7.0 (2.8, 11.2)^**^	0.8 (−3.3, 4.8)^NS^	0.4 (−3.6, 4.4)^NS^	16.4 (9.6, 23.2)^***^	15.9 (9.0, 22.7)^***^	13.9 (6.5, 21.2)^***^	11.7 (4.3, 19.2)^**^
RII	3.1 (1.9, 5.0) ^***^	2.7 (1.7, 4.5)^***^	1.2 (0.6, 2.6)^NS^	1.2 (0.5, 2.5)^NS^	1.7 (1.4, 2.2)^***^	1.7 (1.4, 2.1)^***^	2.1 (1.5, 2.9)^***^	1.9 (1.3, 2.6)^***^
**Sealants**								
SII	−11.7 (− 18.1, −5.4)^***^	−9.3 (−16.4, −2.2)^**^	−7.1 (−15.2, 0.9)^NS^	−5.0 (− 13.2, 3.1)^NS^	−20.9 (− 27.6, − 14.3)^***^	− 18.1 (− 24.8, − 11.4)^***^	− 14.9 (− 23.7, − 6.2)^***^	−14.3 (− 23.1, − 5.4)^**^
RII	0.7 (0.5, 0.8)^***^	0.7 (0.6, 0.9)^***^	0.8 (0.7, 1.0)^NS^	0.9 (0.7, 1.1)^NS^	0.5 (0.4, 0.6)^***^	0.5 (0.4, 0.6)^***^	0.7 (0.5, 0.9)^**^	0.7 (0.5, 0.9)^**^

^a^ Adjusted for age, gender

^b^ Adjusted for age, gender, FTB (frequency of tooth brushing), and RDC (regular dental check-up)

* *P* < 0.05, **P < 0.01, *** *P* < 0.001

## Discussion

This study found that socioeconomic inequality in untreated dental caries and sealant treatment was alleviated for children by an expansion of NHIS coverage in Korea.

The data from KNHANES were analyzed, which is a yearly repeated cross-sectional survey and the data for every three years represent a different wave. The survey continues for three years, which offers the advantage of reflecting fast-changing disease patterns. There could be slight differences every year, meaning that it is imperative that data are handled carefully [20]. This was the reason to analyze this data to compare before and after the policy implementations such as natural experiments. It can be assumed that the changes of Korean people could be found by this sample. Another advantage of this survey is carefully designed to be representative of national non-institutionalized civilians in South Korea. Well-trained dentists took part in this survey which makes the result stronger and reliable. The survey is repeated every year with different samples, not like a cohort. It could be a strength of this survey because the cohort might be impossible to reflect the change of the caries trends with representative samplings. The different characteristics of the fourth and sixth wave samples were applied to the data set with caries and sealant status changes.

After the coverage expansion of dental sealants, the prevalence of untreated dental caries decreased and that of having sealant treatment increased in both children and adolescents. This study also showed an overall increase in dental service usage after the coverage expansion [17]. A similar review on smoking inequality in youth after tobacco control policies concluded that [40] education and information communication led to widening inequalities, while the tobacco price policy reduced socioeconomic inequalities. This is supported by the argument that some public health interventions may increase inequalities [41]. “Upstream” interventions such as reducing price barriers are more likely to have positive effects on alleviating inequalities compared to “downstream” interventions to focus on individual-level factors such as information provided through education. Based on the review of the effects of public health policies on health inequalities, Thomson et al. [42] concluded that two types of oral health interventions had positive effects on inequalities: water fluoridation [43] and a national tooth brushing education campaign [44]. Another study pointed out that dental insurance is an important driver for dental service use, as tackling financial barriers mostly reduces unmet dental needs [17, 45]. US studies to examine the effects of the Children’s Health Insurance Program also reported an increase in sealant treatment, fluoride tablets, and dental visits and a decrease in untreated caries since 1997, especially in children from lower-income households who benefited from free or subsidized school lunch programs [46].

The present study showed differential impacts of coverage expansion on dental health inequality between children and adolescents; the alleviation of inequalities was more salient among children while not among adolescents, especially in untreated dental care. The adolescent group showed decreased prevalence both in the untreated dental caries and sealant. However, the proportional changes in caries were not enough to narrow the gap between the highest and the lowest income groups in adolescents. A difference could be explained as follows. First, inequality may worsen as children grow older, as shown in previous studies [47–50]. A study based on the United Kingdom Millennium Cohort showed relatively narrow in health inequality when the children were younger (aged 3–5) [47]. They were born when the New Labor Government introduced a sustainable strategy to address health inequalities. It could be inferred that the use of preventive dental services may alleviate the disease. Even the same intervention could not have the same effect on older children to alleviate inequality. The second possibility is the ‘inverse care law’ of public health care [51–54]. In the early stage, public health care is used by people with more resources such as information, time, availability, or money, which leads to deepening inequalities. The NHIS dental care coverage in South Korea was implemented in December 2009, and it covered only children aged 6–14 years old for the first molar in permanent dentition with a 30% copayment. In 2012, the coverage was expanded to the second molar and adolescents up to 18 years old in 2013. The adolescents aged 12–18 in the sixth wave were 6–12 years old in the fourth wave (2007–2009). A part of them was not eligible for the service because they were over 14, not qualified for the service until 2013. Later the service was available to all, but some of them already had or had experienced caries, in which dental sealants were no longer applicable. Third, as McLaren pointed out, sometimes the population strategy of prevention will not be effective in narrowing socioeconomic inequalities in health [55]. Preventive services such as sealant treatment could inhibit dental caries, but it is not guaranteed to reduce socioeconomic inequalities in oral health [56]. Based on Taiwan’s National Health Insurance Research Database, Hsu et al. showed that including the preventive provision of fluoride has an effect, but only for specific groups of children who are vulnerable to dental problems [57]. Even though the percentage of children receiving fluoride was increasing, visits for dental caries decreased among those with highly severe caries of primary dentition. One UK study also showed that socioeconomic inequality remained despite no difference in dental health utilization [58]. In the United States, income-related inequality in untreated dental caries among children has been steady over three decades since the 1970s [48, 49]. More salient inequality in dental health observed in developed countries rather than developing ones [4] may be associated with accessibility to dental treatment services as well as sugar consumption [50].

There are several limitations of this study related to the coverage of dental sealants. The NHIS policy changes too often in relation to dental sealant treatment. From the 2010s, the government just agreed on an extension of the coverage provided for dental care to include preventive treatment for the first time. This has not been implemented before, as the government was wary of the possible financial burden. However, their expectations proved to be inaccurate, as fewer than 10% of children received the dental sealant service under the policy coverage every year. Because it was a new approach, the policy went through a transitional phase concerning the extended coverage of dental service in the beginning. This means that the change in policy could have affected the children and adolescents in this study unevenly. Later, in 2017, the government reduced the out-of-pocket payment from 30 to 10% of the total fee. This limitation can be overcome if monitoring of next wave study participants continues.

## Conclusion

This study found that the NHIS coverage expansion of dental care had a positive effect on overall status in dental health among children and adolescents. However, younger children benefited more in terms of inequalities. Our findings indicate that strategies to enhance access to preventive dental services should consider the differential effects for the vulnerable population in terms of socioeconomic status and age from the beginning stage of the policy. The extended coverage policy should continue, focusing on the underprivileged and young children population.

## Data Availability

The datasets used and/or analyzed during the current study are available from the corresponding author on reasonable request.

## Abbreviations

SEP: Socioeconomic Position
Korea: South Korea
NHIS: National Health Insurance Service (NHIS)
KNHANES: Korean National Health and Nutrition Examination Survey
KCDC: Korea Centers for Disease Control and Prevention
WHO: World Health Organization
FTB: Frequency of Tooth Brushing
RDC: Regular dental check-ups
PRs: Prevalence Ratios
SII: Slope Index of Inequality
RII: Relative Index of Inequality
CIs: Confidence Intervals
